# Meprin β contributes to collagen deposition in lung fibrosis

**DOI:** 10.1038/srep39969

**Published:** 2017-01-06

**Authors:** V. Biasin, M. Wygrecka, L. M. Marsh, C. Becker-Pauly, L. Brcic, B. Ghanim, W. Klepetko, A. Olschewski, G. Kwapiszewska

**Affiliations:** 1 Ludwig Boltzmann Institute for Lung Vascular Research, Graz, Austria; 2 Department of Biochemistry, Faculty of Medicine, University of Giessen Lung Center, Giessen, Germany; 3 German Centre for Lung Research (DZL), Giessen, Germany; 4 Institute of Biochemistry, Unit for Degradomics of the Protease Web, University of Kiel, Kiel, Germany; 5 Institute of Pathology, Medical University of Graz, Graz Austria; 6 Department of Surgery, Division of Thoracic Surgery, Medical University of Vienna, Vienna, Austria; 7 Department of Physiology, Medical University of Graz, Graz, Austria

## Abstract

Lung fibrosis is a severe disease characterized by epithelial cell injury, inflammation and collagen deposition. The metalloproteases meprinα and meprinβ have been shown to enhance collagen maturation and inflammatory cell infiltration via cleavage of cell-cell contact molecules; therefore we hypothesized that meprins could play a role in lung fibrosis. An exhaustive characterization of bleomycin-treated meprinα, meprinβ and the double meprinsαβ knock-out (KO) with respective wt-littermates was performed by using several different methods. We observed no difference in lung function parameters and no change in inflammatory cells infiltrating the lung between wt and all meprins KO mice after 14 days bleomycin. No difference in epithelial integrity as assessed by e-cadherin protein level was detected in bleomycin-treated lungs. However, morphological analysis in the bleomycin-treated mice revealed decrease collagen deposition and tissue density in meprinβ KO, but not in meprinα and meprinαβ KO mice. This finding was accompanied by localization of meprinβ to epithelial cells in regions with immature collagen in mice. Similarly, in human IPF lungs meprinβ was mostly localized in epithelium. These findings suggest that local environment triggers meprinβ expression to support collagen maturation. In conclusion, our data demonstrate the *in vivo* relevance of meprinβ in collagen deposition in lung fibrosis.

The astacin metalloproteases, meprin α and meprin β are proteases involved in cleavage of growth factors and extracellular matrix proteins^1^. They have been shown to cleave the C and N-terminal prodomains of pro-collagen I and III, leading to collagen maturation^2^. In accordance, meprin α and meprin β knock out (KO) animals showed a reduced dermal collagen deposition and impaired arrangement of the collagen fibrils which decreased tensile strength of the skin^3^. Meprin α and meprin β were found to be elevated in fibrotic skin (called keloids)^4^. Furthermore meprin β was the most upregulated gene in the lungs of fra-2 over-expressing mice, a genetic mouse model which possesses several features of idiopathic pulmonary fibrosis (IPF)^5^,^6^. Idiopathic pulmonary fibrosis (IPF) is a rare and severe interstitial lung disease with unknown aetiology and poor prognosis^7^. The 5-year survival rate is approximately 10–15% from the time of diagnosis^8^. IPF is characterized by chronic alveolar epithelial injury, which results in massive fibroblast proliferation and extracellular matrix (ECM) deposition and thereby scarring of the lung tissue^9^,^10^. A plethora of mediators such as growth factors, cytokines, chemokines, and matrix metalloproteinases (MMPs) have been implicated in the disease progression^11^. MMPs lead to basement membrane disruption and thus invasion of fibroblasts to alveolar space where they proliferate and produce ECM proteins such as collagens^12^,^13^,^14^. The cellular and tissue localization of MMPs and their inhibitors (tissue inhibitor of metalloproteases, TIMPs) is crucial for ECM homeostasis as it determines degradation and/or deposition of ECM proteins and therefore the heterogeneity of the fibrotic disease^15^. In lung fibrosis, collagen deposition is part of a tissue healing process which is triggered by the injury of the epithelial cells. The disruption of the epithelial layer integrity can enhance inflammatory cell infiltration and in turn worsen the fibrotic process^16^. Meprins have been shown to cleave cell-cell contact molecules on epithelial cells such as E-cadherin^17^ and occludin^18^. This cleavage favours inflammatory cell infiltration^1^, a process which has been shown to be influenced by meprins, as meprins KO mice exhibit deficiency in cell extravasation^19^.

These findings suggest that meprins can be important in the onset of fibrosis, contributing to epithelial layer disruption, inflammatory cell infiltration and collagen maturation. However, their role in lung fibrosis has not been investigated so far. Hence, the aim of the current study is to delineate the contribution of meprins to the onset of pulmonary fibrosis and to investigate potential underlying molecular mechanism.

## Results

### Meprins are expressed in inflammatory and epithelial cells of human lungs

**Figure 1:**
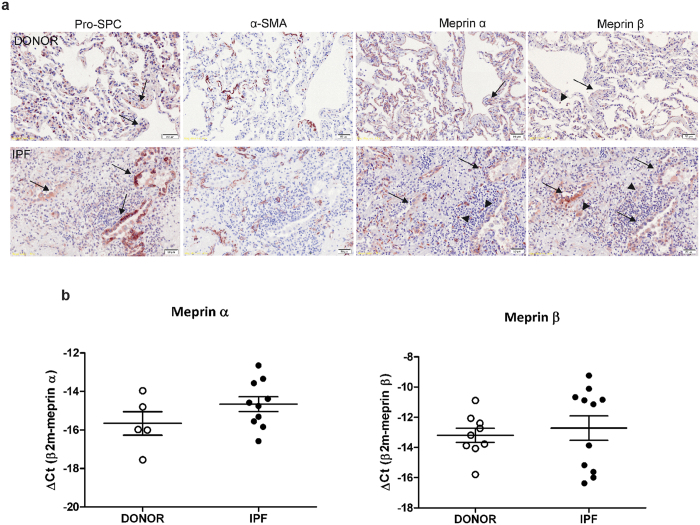
Meprins are expressed in inflammatory and epithelial cells of human lungs. (**a**) Serial slides staining for pro-surfactant protein-C (pro-SPC), alpha-smooth muscle actin (α-SMA), meprin α, and meprin β in lungs from donor (n = 4) and IPF (n = 4). Arrows and arrowheads indicate staining of meprins in epithelial cells and inflammatory cells respectively. Scale bars show 50 μm. (**b**) mRNA expression level of meprin α and meprin β in lung homogenate from donor (n = 10) and IPF (n = 12). Every dot correspond to a single donor or IPF patient, fewer n number than 10 (donor) and 12 (IPF) indicate non detectable samples.

We first assessed the localization of both meprins α and β by immunohistochemical staining in human donor and IPF lungs. We observed that meprins expression localized to epithelial and inflammatory cells (Fig. 1a). To confirm the epithelial cell localization of meprins we performed immunohistochemical staining on serial slides with pro-surfactant protein C (pro-SPC, marker for alveolar type II epithelial cells) and smooth muscle actin (SMA; marker for smooth muscle cells and myofibroblasts). The staining revealed that meprins are mostly localized to pro-SPC positive cells (Fig. 1a, arrow) and inflammatory cells (Fig. 1a, arrowhead), while myofibroblasts were negative for meprins (Fig. 1a). PCR analysis revealed that meprins are expressed at very low levels in human lungs; however meprin β level is higher compared to meprin α (as indicated by their ΔCt values) (Fig. 1b). We did not detect any substantial change of meprins expression in lung homogenate of IPF in comparison to donor samples (Fig. 1b). All immunohistochemistry controls are shown in Supplementary Figure S1.

### Meprin β is regulated by TGF-β1

**Figure 2:**
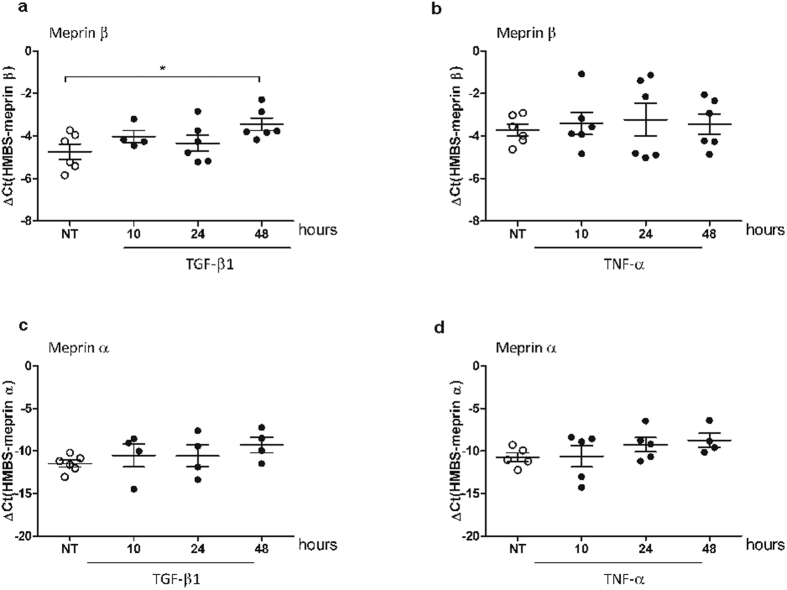
Meprin β is regulated by TGF-β1 in epithelial cells. mRNA expression level of meprin β and meprin α upon TGF-β1 (10 ng/ml) (**a**,**c** respectively) and TNF-α (1 ng/ml) (**b,d** respectively) stimulation on A549 cells for the indicated time points (*p < 0.05).

As analysis of total lung homogenate may mask cell specific changes in meprin expression, we examined how pro-fibrotic and pro-inflammatory factors could directly affect their expression in epithelial cells. Following stimulation of A549 cells with TGF-β1, we observed that meprin β, but not meprin α, was upregulated upon 48 h (Fig. 2a,c), suggesting an indirect regulation upon activation of TGF-β1 pathway. TNF-α and short time course with TGF-β stimulation did not influence the expression of either meprins (Fig. 2b,d and Supplementary Figure S2).

### Meprins are important for epithelial monolayer integrity

**Figure 3:**
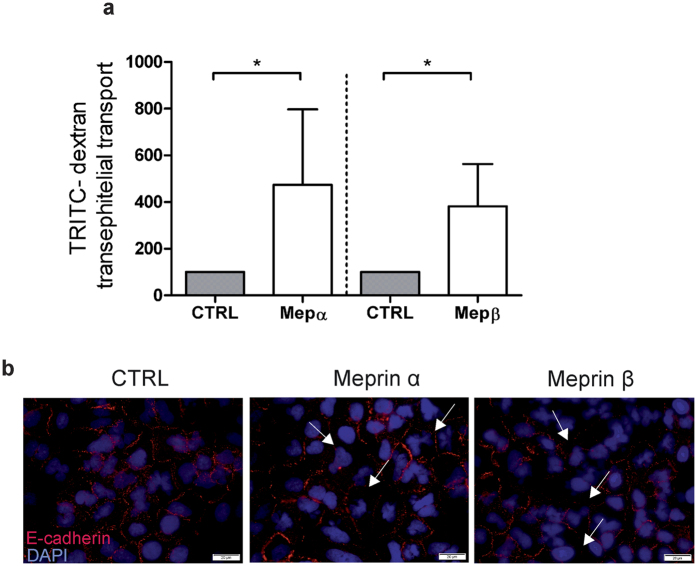
Meprins are important for epithelial monolayer integrity. (**a**) Addition of 40 nM activated human recombinant meprin α and meprin β increased TRITC-dextran permeability of A549 cells (*p < 0.05). (**b**) Immunofluorescence picture of E-cadherin staining on A549 cells stimulated with vehicle (CTRL), human recombinant activated meprin α and meprin β. Arrows indicate interruption of E-cadherin staining. Stimulation was performed 4 times in duplicate (*p < 0.05). Scale bars show 20 μm.

Meprins have previously been shown to cleave E-cadherin, a cell-cell contact molecule, in colon and kidney epithelial cells^17^. Disruption of the epithelial integrity is considered to be an early event in lung fibrosis^16^; therefore we tested whether meprins can alter the A549 cell monolayer *in vitro.* Our permeability assay showed that meprin α and meprin β treatment resulted in a significant increase of cell permeability (Fig. 3a). Positive controls are depicted in Supplementary Figure S3. Immunofluorescence staining confirmed the weaker signal of E-cadherin (Fig. 3b), indicating that meprins can be involved in cell-cell contact protein degradation and thereby in the disruption of epithelial layer integrity.

### Meprin β localisation changes upon bleomycin treatment

**Figure 4:**
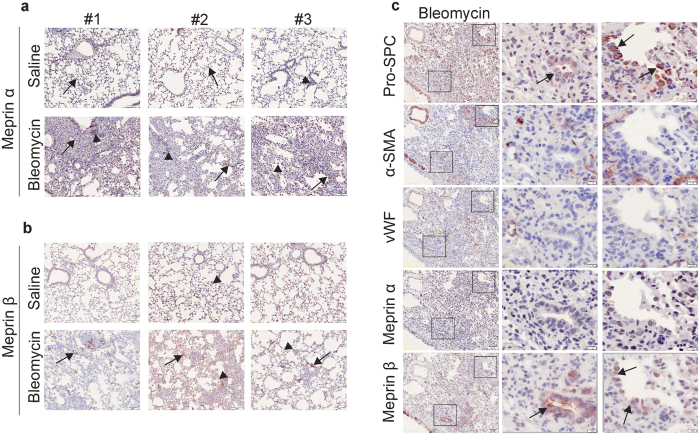
The localisation of Meprin β but not Meprin α changes upon bleomycin treatment. Representative pictures of immunohistochemical staining for (**a**) meprin α and (**b**) meprin β in mouse saline (n = 4) and bleomycin treated lungs (n = 5), after 14 days bleomycin injection. Arrows point at meprin staining in epithelial cells, while arrowheads point at meprin staining in inflammatory cells. Scale bars show 50 μm. (**c**) Representative pictures of immunohistochemical staining in serial slides for pro-surfactant protein C (pro-SPC), α-smooth muscle actin (α-SMA), von willebrand factor (vWF), meprin α and meprin β. Arrows point at positive staining for meprin β and pro-SPC. Scale bars show 50 μm in the overview picture and 10 μm in the zoomed area.

In order to assess meprins expression in the bleomycin model of lung fibrosis, we performed immunohistochemical staining. Similar to the human data, meprin α and meprin β were localized mostly to epithelial cells and to inflammatory cells (Fig. 4a,b, arrows and arrowheads respectively). Interestingly, after 14 days bleomycin administration, the localization of meprin β was restricted to specific areas of the lung parenchyma (Fig. 4b, arrow). To verify the cellular localization of meprins, we performed serial slide staining with pro-SPC, α-SMA and von Willebrand Factor (marker for endothelial cells). In bleomycin treated mice, we observed that meprin β is mostly express in pro-SPC positive cells (Fig. 4c). Meprin α showed a ubiquitous expression in the lung, irrespectively of the treatment and a very weak staining indicating low expression (Fig. 4c).

### Lack of meprins do not result in improved lung function in bleomycin treated mice

**Figure 5:**
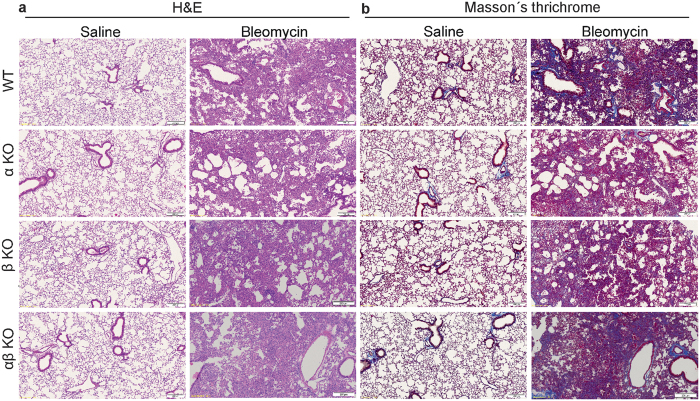
Lack of meprins do not influence bleomycin induced phenotype. (**a**) H&E and (**b**) Masson’s Trichrome staining of lung section from wt littermates, meprin α, meprin β, meprin αβ KO mice after 14 days saline or bleomycin treatment. Scale bars show 200 μm.

**Figure 6:**
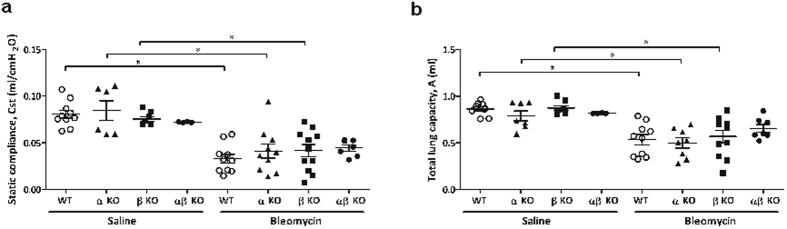
Lung function is not preserved in meprins KO mice upon bleomycin treatment. Assessment by flexivent of (**a**) static compliance (Cst) and (**b**) total lung capacity (A) in wt littermates, meprin α, meprin β meprin αβ KO mice after 14 days saline or bleomycin treatment (*p < 0.05).

To test whether meprins have a role in fibrosis development *in vivo*, we subjected wt, meprin α, β and αβ KO mice to bleomycin. Histological analysis, revealed no apparent differences between wt mice and meprins KO mice (Fig. 5). Measurement of the lung function of the mice, revealed a decrease of compliance (Fig. 6a) and total lung capacity (Fig. 6b) upon bleomycin treatment, however no differences were observed between bleomycin treated wt littermates and meprins KO mice irrespectively of genotype.

### Absence of meprins does not prevent E-cadherin loss in bleomycin challenged mice

**Figure 7:**
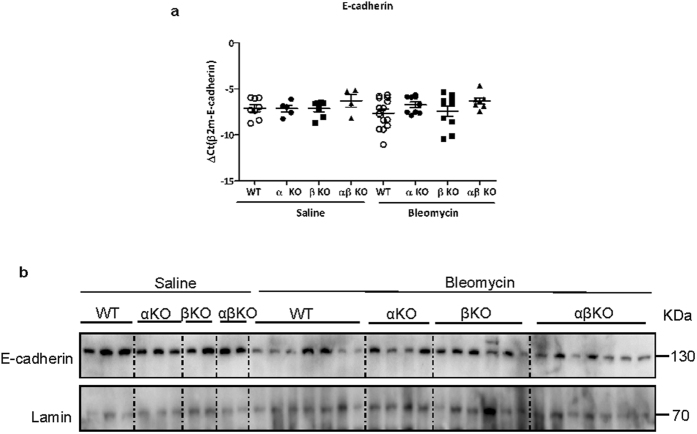
Absence of meprins does not prevent E-cadherin loss in bleomycin treatment mice. (**a**) mRNA and (**b**) protein level of E-cadherin in lung homogenate from meprin α, meprin β meprin αβ KO and wt littermates mice after 14 days saline or bleomycin treatment (*p < 0.05). Western blots pictures have been cropped and only Lamin A is shown as housekeeping protein.

As it has been reported that epithelial cells downregulate E-cadherin expression in fibrosis^20^,^21^ and meprins can influence its abundance, we analysed the expression of E-cadherin in *in vivo* settings upon saline and bleomycin treatment. No changes in E-cadherin mRNA level were observed between saline and bleomycin treated mice, irrespectively of the genotype (Fig. 7a). Western blot analysis revealed a decrease of E-cadherin in bleomycin wt mice in comparison to wt saline treated mice (Fig. 7b); however we did not observe any apparent difference of E-cadherin protein level between different genotypes (Fig. 7b and Supplementary Figure S4).

### Meprins do not influence number and composition of inflammatory cells in the bleomycin treated lungs

**Figure 8:**
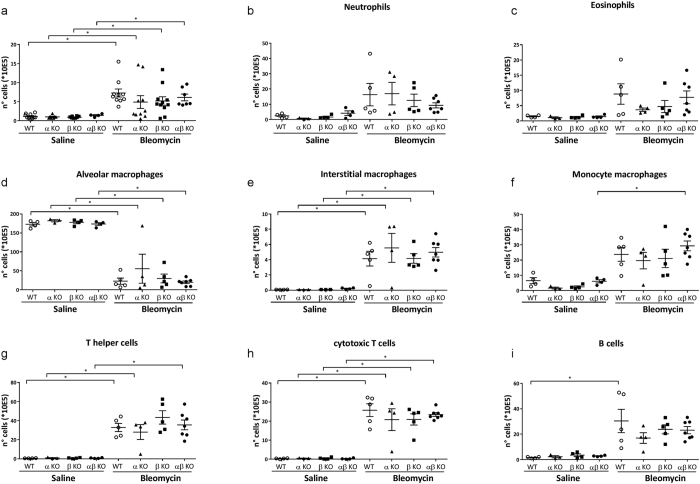
Meprins do not influence inflammatory infiltrate after 14 days bleomycin treatment. (**a**) Representation of the cell counts from broncho-alveolar lavage fluid (BALF) from meprin α, meprin β meprin αβ and wt littermates mice after 14 days saline or bleomycin treatment (*p < 0.05). FACS analysis performed from the BALF from meprin α, meprin β meprin αβ KO and wt littermates mice after 14 days saline or bleomycin treatment (*p < 0.05). Detection of (**b**) Neutrophils (**c**) Eosinophils, (**d**) Alveolar macrophages, (**e**) Interstitial macrophages, (**f**) Monocyte macrophages, (**g**) T helper cells, (**h**) cytotoxic T cells and (**i**) B-cell.

Meprins have been also shown to influence the infiltration of inflammatory cells in the intestinal tissue^22^. Inflammatory cells have been shown to be an important component of bleomycin induced lung fibrosis^23^. Hence, we analysed the inflammatory cells in the BALF of saline and bleomycin wt, meprin α, meprin β and meprin αβ KO mice. As depicted in Fig. 8a, we observed an increase in inflammatory cells in the BALF of bleomycin treated mice in comparison to saline; however deletion of meprins did not affect this increase. To rule out the possibility that the inflammatory infiltrate composition is different, we performed FACS analysis from the BALF. Although we observed several changes such as increase of macrophages, T and B-cells (Fig. 8b–i) due to bleomycin application in wt mice in comparison to wt saline mice; we could not detect any significant change among different genotypes (Fig. 8b–i). These results suggest that meprins do not influence the inflammatory composition in the lung after 14 days of bleomycin treatment.

### Meprin β KO mice have less collagen deposition in comparison to wt littermates

**Figure 9:**
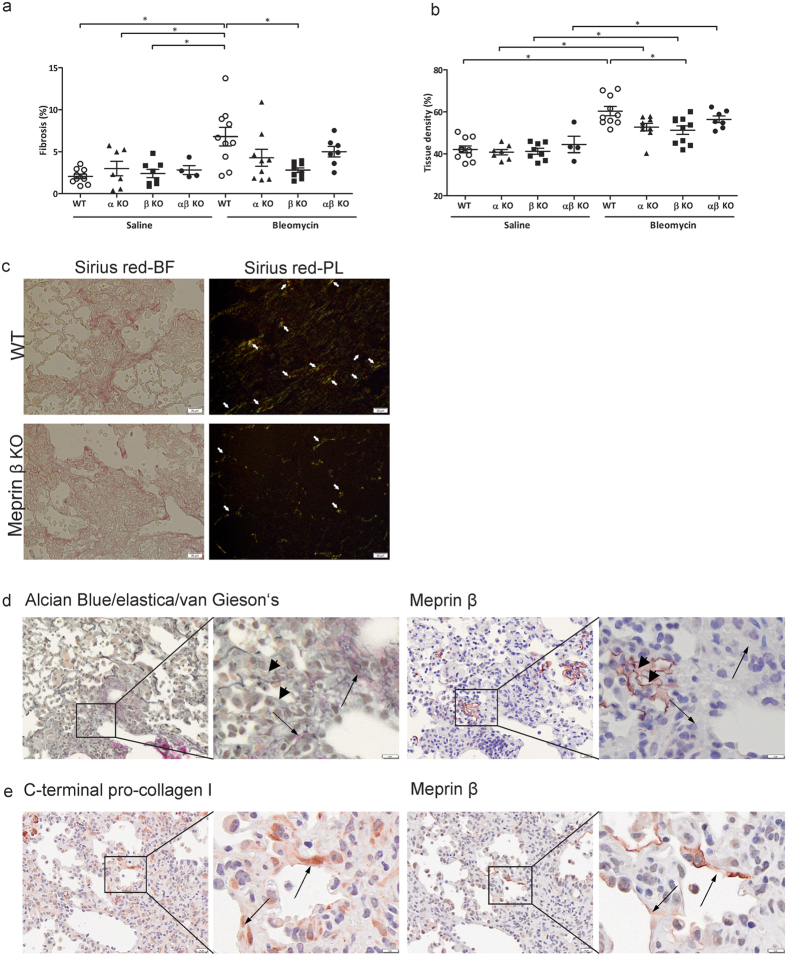
Meprin β KO mice have less collagen deposition in comparison to wt littermates and meprin β localize in region rich with immature collagen. Quantification of (**a**) fibrosis and (**b**) tissue density from masson’s trichrome staining slides of meprin α, meprin β meprin αβ KO and wt littermates mice after 14 days saline or bleomycin treatment (*p < 0.05). (**c**) Representative pictures of Sirius red staining under bright field (BF) and polarized light microscopy (PL) of bleomycin treated wt and meprin β KO mice. Arrows point at region with collagen. Scale bars show 20 μm. (**d**) Alcian blue/elastica/van Gieson’s staining and meprin β in serial slides from lung wt mice after 14 days bleomycin treatment. Arrows point at mature collagen (pink) while arrowshead point at immature collagen (greenish grey). Scale bars show 50 μm in the overview picture and 10 μm in the zoomed area. (**e**) Representative pictures of immunohistochemical staining in serial slides for C-terminal-pro-collagen I and meprin β. Arrows point at co-localization of meprin β and C-terminal pro-collagen I. Scale bars show 50 μm in the overview picture and 10 μm in the zoomed area.

Meprins can also facilitate collagen maturation^3^; we therefore investigated whether the collagen amount is altered in meprins KO mice in comparison to wt mice upon bleomycin treatment. Quantification of collagen deposition was performed on masson’s trichrome stained slides. As expected, bleomycin treatment increased the amount of collagen in comparison to saline mice (Fig. 9a). Interestingly, we observed that meprin β KO mice upon bleomycin treatment had a lower amount of collagen in the lung in comparison to bleomycin treated wt littermates (Fig. 9a). The decreased collagen deposition in meprin β KO mice was concomitant with lower tissue density in comparison to wild type mice (Fig. 9b). Additionally, Sirius red stained slide subjected to polarized light confirmed a diminished birefringence signal in bleomycin treated meprin β KO mice compared to wt animals challenged with bleomycin suggesting a decrease collagen amount (Fig. 9c and Supplementary Figure S5). Expression of collagen I and III was elevated upon bleomycin in comparison to saline, but no differences were observed between different genotypes, indicating that post-translational modification is responsible for the reduction in collagen content in meprin β KO mice (Supplementary Fig. S6a,b). Another protease which belongs to meprin family, bone morphogenetic protein-1 (BMP-1), can also enhance maturation of collagens^24^. We therefore determined whether the expression of BMP-1 was enhanced in KO mice and could potentially compensate for the absence of meprins. However, we did not observe any difference in the expression of BMP-1 in saline or bleomycin treated meprins KO mice (Supplementary Figure S7).

### Meprin β localizes in regions of the lung with immature collagen

To better assess the role of meprins with collagen maturation, we performed Alcian Blue combined with Elastica and van Gieson’s staining in order to visualize the mature and immature collagen. Serial slides were stained for meprin β to assess possible co-localization of immature collagen with meprin β. Interestingly we observed that regions with mature collagen (indicated with normal arrow, purple staining) showed weak or absence of meprin β immunoreactivity, while regions with immature collagen (indicated with arrowheads, light greenish grey staining) showed positivity for meprin β (Fig. 9d and Supplementary Figure S8a). Additionally, staining of C-terminal pro-collagen I and meprin β in serial sections revealed that meprin β localized with region positive for pro-collagen I (Fig. 9e and Fupplementary Figure S7b). These results cumulatively confirms *in vivo* the role of meprin β in maturation of collagen.

## Discussion

In this study we have investigated the role of meprin α and meprin β proteases in development of lung fibrosis. We could show in *vitro* that meprin α and meprin β are involved in injury of the epithelial layer integrity and that they are mostly expressed by epithelial cells. Additionally, meprin β but not meprin α contributes to collagen deposition *in vivo*. Several other metalloproteinases (MMP2, MMP3, MMP7), have been shown to be elevated in BALF of IPF patients and to correlate with the decline of the lung function^25^,^26^,^27^. However, it is becoming clearer that their expression level has to be considered together with their cellular and tissue localization^28^. MMP-1 was found to be elevated in IPF patients, even though its main function is degradation of collagen^27^,^29^. Although it sounds paradoxical, further analysis revealed that MMP-1 is upregulated in reactive epithelium and not in the lung interstitium where the collagen is deposited^27^. In this study we could clearly show that meprins are expressed by epithelial and inflammatory cells of the mouse and human lung. Injury of the epithelial layer is an early event in lung fibrosis^10^,^16^, which is characterized by loss of cell-cell contact molecules, such as E-cadherin^30^. In line with previous studies^17^,^18^, we could show that meprins are involved in disturbance of the lung epithelial cell layer integrity *in vitro. In vivo*, we did not observe any apparent differences in E-cadherin protein levels in meprins KO mice in comparison to wt. As E-cadherin is also degraded by other proteases^31^,^32^, it is likely that other molecular pathways take part in E-cadherin cleavage in bleomycin induced lung fibrosis. Degradation of epithelial cell-cell contact protein is followed by inflammatory cell recruitment in the tissue, which influences the progression of fibrosis^33^. Meprins KO animals have been shown to have deficient extravasation of inflammatory cells to the intestinal mucosa in inflammatory bowel disease mouse model^22^. In our study we could not observe any difference in number and type of cells infiltrating the lung, suggesting that meprins are not essential for the cell extravasation in the lung upon bleomycin treatment at the investigated time point.

The infiltrated inflammatory cells release several pro-fibrotic chemokines which promote collagen production^28^,^34^. Here we could show *in vivo* that bleomycin treated meprin β KO mice accumulate less collagen than bleomycin treated wt littermates. These findings suggest a potential role of the metalloprotease meprin β in collagen deposition in lung fibrosis. Interestingly, we did not observe any difference in lung function parameters in bleomycin treated meprin β KO mice in comparison to wt. As the decline of lung function in pulmonary fibrosis is a result of the epithelial injury, inflammatory influx and collagen deposition^35^, we could speculate that in meprin β KO mice change in collagen amount, but not in inflammation, was insufficient to improve the lung function parameters. Interestingly, in bleomycin treated mice and in human lung fibrosis, meprin β displayed a well localized and definite expression pattern to epithelial cells. The localization of meprin β on epithelial cells and the reduction of collagen in the meprin β KO lung, suggest that meprin β in pathological condition could take part to collagen maturation as a protection mechanism against the epithelial cell injury. This was further supported by the change of meprin β localization in saline and bleomycin condition, suggesting a switch on/off mechanism of meprin β expression depending on the need of producing collagen. This mechanism might fuel the perpetual injury and collagen maturation cycle which contribute to the progression of the disease^36^. To support this idea, we showed that immature collagen and pro-collagen I localized in close proximity to meprin β. However, in regions of mature collagen, meprin β was found to be very weakly or not expressed, indicating that meprin β expression is very dynamic and is regulated depending on the need of the surrounding environment. Interestingly, the two meprins have different localization which suggests different behaviour in pathological condition. Although in human lung samples both meprins are expressed and *in vitro* studies have shown that both meprins are capable of maturating collagen I and III^2^, our animal experiments showed that meprin β, but not meprin α, has *in vivo* relevance and contributes significantly to collagen maturation in the lung. Broder *et al*. have previously reported that meprin α and meprin β KO mice have less collagen in the skin and therefore less tensile strength^1^. The different level of meprin α expression between skin and lung could explain the lack of phenotype in meprin α KO mice. Both meprins are weakly expressed in the mouse lungs; particularly with meprin α being often at the detection limit (data not shown). As meprin α is very low expressed in mouse lung, we would expect the αβ KO mice to have a similar phenotype as meprins β KO mice in term of collagen amount. Interestingly, no difference in collagen amount has been noticed in meprins αβ KO mice, which could be explained with other proteases being activated as a compensatory mechanism. Several proteases, such as BMP-1^24^, ADAMTSs^37^ or furin like pro-protein convertase^38^ are involved in pro-collagen processing. Even though we did not observe any difference in the mRNA expression level of BMP-1, we cannot exclude that BMP-1 or other protease´s activity is more pronounced in the αβ KO mice.

Taken together our study assessed the role of meprins in lung fibrosis development. We showed that both meprin α and meprin β are involved in disturbance of epithelial layer integrity *in vitro*, which might represent one of the trigger mechanisms for the fibrosis development. Importantly, we could prove that meprin β is involved in collagen deposition *in vivo* in the lung of bleomycin treated mice and it localized in region with immature collagen. This suggests that meprin β can favour the progression of the fibrotic process enhancing collagen processing and deposition.

## Material and Methods

### Human material

Lung tissues were collected from idiopathic pulmonary fibrosis (IPF) patients who underwent lung transplantation at the Department of Cardiothoracic Surgery, Medical University of Vienna, Austria (Director W. Klepetko). The applied protocol and the usage of the tissue were approved by the institutional ethics committee (EK 976/2010) of the Medical University of Vienna and patients inform consent was obtained before lung transplantation. Downsized non-tumorous non-transplanted donor lungs served as controls. All experiments with human material were performed in accordance with the relevant guidelines and regulations. Samples were fixed in 4% (m/v) paraformaldehyde for histology and immunohistochemistry.

### Animals

Generation of meprin α and β KO mice were previously described^39^,^40^ and they were obtained from Prof. Becker-Pauly (Department of Biochemistry, University of Kiel) and Prof. Gunnar (Department of Biochemistry, University of Gothenburg, Sweden). The meprin αβ double KO mice were generated by crossing meprin α KO mice and meprin β KO. Meprins KO mice (α, β and αβ) and their wild-type littermates were kept under conventional conditions. All animal experiments were approved by the local authorities (Austrian Ministry of Education, Science and Culture) (BMWF-66.010-0038-II-3b-2013) and were performed in accordance with relevant guidelines and regulations.

### Bleomycin administration

Mice (wt, α KO, β KO and αβ KO) were anesthetized with isolfuorane 2–2.5% and intra-tracheal administration of bleomycin (3 U/kg b.w) (Sigma, Vienna, Austria) or saline solution (0.9% w/v NaCl) was applied with a MicroSprayer® Aerosoliser (Penn-Century. Inc, PA, Pennsylvania, USA). Mice were closely monitored till they completely recovered from anaesthesia. Bleomycin administration was performed once and the lungs were collected after 14 days.

### Lung function measurement

Analysis of the respiratory mechanics was performed using FlexiVent system (SCIREQ, Paris, France). Mice were intraperitoneal anesthetized with ketamine (15 mg/ml) and xylazyne (2 mg/ml). Tracheotomy was performed and mice were connected to the FlexiVent system to perform measurement of the respiratory mechanics. Mice were ventilated at a rate of 150 breaths/min with a positive end-expiratory pressure (PEEP) of 3 cmH_2_O. After stable ventilation was achieved three cycles of deep inflation, snapshot, prime-wave (quickprime-3) and pressure-volume loop (PVs-P) perturbations were applied for each mouse. All perturbations were automatically adjusted to the body weight of the mouse by the Flexiware software 7.6. Three measurements were averaged to give the represented the value of one biological sample. After measurement, the mice were euthanized and the blood, broncho-alveolar lavage fluid (BALF) and lungs of all animals were collected for molecular and histological analysis.

### FACS analysis

BALF samples were subjected to cytometric analysis. All inflammatory cells were first selected using a CD45^+^ gate. Staining for neutrophils (CD11b^+^, CD11c^−^, Gr-1^+^), alveolar macrophages (CD11b^low^, CD11c^+^, SiglecF^+^), dendritic cells (CD11b+, CD11c^+^, CD24^+^ MHC-II^high^), T helper cells (CD3^+^, CD4^+^), cytotoxic T cells (CD3^+^, CD8^+^), B cells (CD19^+^), eosinophils (CD11b^+^, CD11c^−^, SiglecF^+^), interstitial macrophages (CD11b^+^, CD64^+^ SiglecF^−^) and Ly6C/G^+^ monocyte macrophages (CD11b^+^, CD64^−^, Ly6C/G^+^) was performed and assessed by a LSRII flow cytometer^41^. Analysis was carried out with the FACSDiva software (both from BD Biosciences, Heidelberg, Germany).

### Cell stimulation

A549 cells were purchased from ATCC (Wesel, Germany) and were cultured in DMEM F/12 medium with 10% FCS and 1% Penicillin/Streptomycin. Stimulation of A549 cells was performed after 12 h starvation (DMEM F/12 medium with 1% Penicillin/Streptomycin without FCS) with TGF-β1 (10 ng/ml, R&D Systems Inc Minneapolis, MN, USA) and TNFα (1 ng/ml, eBioscience, San Diego, USA) for the indicated time points.

### Meprin α and meprin β activation

Human latent recombinant meprin α and meprin β were purchased from R&D. Stimulation of A549 cells was performed after catalytic activation of meprins; latent recombinant meprins (40 nM) were incubated with trypsin (ratio trypsin:meprin of 1:20) for 30–45 min for meprin α and 60 min for meprin β at 37 °C. Trypsin was then inhibited with 10 fold excess soybean trypsin inhibitor (STI) for 20 min at RT. Control reaction containing trypsin and STI without meprins was used as a negative control.

### Permeability assay

A549 cells were seeded and grown until they formed a confluent monolayer on semi-permeable inserts (Corning, Tewksbury MA, USA). After 12 h starvation, cells were incubated with control solution, recombinant meprin α, recombinant meprin β and DMSO. After 1 h treatment, TRITC-labelled dextran (Sigma) was added to the upper compartment of the insert. The fluorescence from the bottom well was measured with a fluorescent plate reader (FLUOstar Omega, BMG LABTECH, Ortenberg, Germany) (Exc 544 nm, Em 590 nm). TRITC-dextran was added on a well without cells (called no monolayer, nML) to give the maximum fluorescent signal. This signal was used for gain adjustment of the detector sensitivity, in order to achieve the correct sensitivity.

### Immunohistochemistry and tissue staining

Formalin-fixed paraffin embedded lung tissues were cut to 2.5 μm thick sections and stained with either Haemalaun-eosin, Masson’s trichrome, used for immunohistochemistry, Sirus red or Alcian Blue combined with Elastica and van Gieson’s staining.

Immunohistochemistry was performed using ImmPACT ^TM^ VIP Kit (Vector Laboratories, Burlingame, CA, USA) according to the manufacturer’s instructions. The following dilutions of primary antibodies were used: for human samples anti-meprin β (1:100; AF2895 R&D), anti-meprin α (1:500, AF3220, R&D) anti-proSPC (1:1000; AB3786 Millipore, Vienna, Austria), anti-SMA (1:100; PA5–19465 Thermo Scientific). For mouse samples anti-meprin β (1:100; AF3300 R&D), anti-meprin α (1:2000; from Prof. C.Becker Pauly), anti-vWF (1:500, A0082, DAKO, Vienna, Austria), anti-ProSPC, anti-SMA (as above) and anti- α1 C-terminal pro-collagen (1:500 from Prof. C.Becker Pauly) were used. Negative controls were performed with the omission of the first antibody. Slides were scanned and images were acquired with a Virtual Slides Microscope and Olyvia Software (both from Olympus, Vienna, Austria).

To determine tissue density and collagen content Masson’s trichrome slides were subjected to semi-automated image analysis software Visiopharm (Hoersholm, Denmark). Collagen content was calculated as a percentage of the total analysed area, as previously described^42^. Tissue density was calculated as a percentage of total tissue to the total analysed area. In both analysis the whole left and right lung were considered.

Sirius Red staining was performed according to manufactory’s instruction. The stained slides were subjected to bright field and polarized light (Olympus) for qualitative analysis of the collagen using the microscope Olympus BX51.

Alcian Blue combined with Elastica and van Gieson’s staining was performed on lung section from bleomycin treated wt mice. Previous studies have already shown the capability of identification of immature collagen with acid fuchsin^43^,^44^. This staining highlights: nuclei (dark brown), elastic fibres (blue-violet), mucus (blue), collagens (red), muscle (ochre) and immature collagen (greenish grey).

### Immunofluorescence

For immunofluorescence analysis, A549 grown on 8-well chamber slides were fixed for 20 min in cold methanol at −20 °C and rinsed five times in PBS. Afterwards, the cells were permeabilized for 5 min with 0.5% TritonX-100 in PBS at RT, washed twice with PBS and incubated overnight at 4 °C with anti-E-cadherin (1:500; U3254, Sigma). Cells were then washed three times with PBS and incubated with anti-rat conjugated with Alexa Fluor®Dye 555 (Thermo Scientific) for 1 h at RT in the dark. Cells were washed three times in PBS and mounted with fluorescence vectashield mounting medium with 4′,6-diamidino-2-phenylindole dihydrochloride (DAPI) for the counterstaining (Vector). For microscopic inspection, an Olympus fluorescence microscope was used.

### Statistical analysis

Data are presented as mean ± standard error of the mean (SEM) for all experiments. Statistical analysis was performed in GraphPad Prism 5 software. For comparison of two groups, unpaired t-test was carried out. Differences between more than two groups were assessed by one-way ANOVA followed by Bonferroni post-hoc test. Differences in time course stimulation experiments were assessed with one-way ANOVA followed by Dunnet post hoc test. For the permeability assay, T-test with wilcoxon matched pair signed rank was used. Significance is indicated by an asterisk on the graphs. A p value less than 0.05 was considered significant for all analysis. All experiments were designed with matched control conditions to enable statistical comparison.

## Additional Information

**How to cite this article**: Biasin, V. *et al*. Meprin β contributes to collagen deposition in lung fibrosis. *Sci. Rep.* **7**, 39969; doi: 10.1038/srep39969 (2017).

**Publisher's note:** Springer Nature remains neutral with regard to jurisdictional claims in published maps and institutional affiliations.

## Supplementary Material

Supplementary Information
