# Supplementary material for: Meprin β contributes to collagen deposition in lung fibrosis
Source: Sci Rep. 2017 Jan 6;7:39969. doi: 10.1038/srep39969 (PMC5216360; doi:10.1038/srep39969)
Supplement: Supplementary Information [file srep39969-s1.doc]

**Meprin β contributes to collagen deposition in lung fibrosis**

Biasin V, Wygrecka M, Marsh LM, Becker-Pauly C, Brcic L, Ghanim B, Klepetko W, Olschewski A, Kwapiszewska G*.

**Supplementary materials and methods**

**BALF collection**

BALF was collected via the trachea by slow injection and recollection of 1ml BAL solution (PBS, 2mM EDTA and protease inhibitor (Pierce, Waltham, MA, USA)).

**RNA isolation and real-time PCR**

Total RNA from human and mouse lung tissue was isolated using Trizol reagent (Thermo Scientific, Waltham, MA USA) according to manufacturer’s instruction. Total RNA from cells was isolated with the peqGOLD isolation kit (PeqLab, Erlangen, Germany). Quantity of RNA was determined by absorbance at 260 and 280 nm (Nanodrop). Total RNA was reverse transcribed using iScript kit (BioRad, Hercules, CA, USA) according to manufacturer’s instructions. Real-time PCR was performed using a LightCycler 480 (Roche, Vienna, Austria). The PCR reactions were set up using QuantiFast SYBR PCR kit (Qiagen, Hilden, Germany). Cycling conditions were as follows: 5 min at 95°C, [5 seconds at 95°C, 5 seconds at 60°C, and 10 seconds at 72°C] x45 cycles. Due to the non-selective double strand DNA binding of the SYBR®Green I dye, melting curve analysis and gel electrophoresis were performed to confirm the specific amplification of the expected PCR product. Primer sequences are provided in supplementary table 1.

**Protein isolation and western blotting**

Proteins were isolated from mouse lung tissue with liquid nitrogen. The mouse lung tissue was crashed to powder with mortar and pestle in liquid nitrogen. The powder was dissolved in RIPA buffer (Sigma) supplemented with protease and phosphatase inhibitors (Pierce, Waltham, MA, USA). Samples were vortexed and incubated for 30 min on ice, before centrifugation for 15 min at 16.000 g at 4°C and supernatants collected. Extracts were separated on a SDS polyacrylamide gel, followed by electro-transfer to a PVDF membrane. After blocking with 5% BSA in TBS-T buffer (150mM NaCl, 25mM Tris-HCl, ph 7.5, 1% Tween 20), the membrane was incubated overnight at 4°C with one of the following antibodies: anti E-cadherin (rat, 1:1000, Sigma), and anti-lamin (rabbit, 1:2000, Cell Signalling, Leiden, Netherlands). After 1h incubation with peroxidase-labelled secondary antibody (Pierce), proteins were detected using ECL Prime Kit (GE Healthcare, Vienna, Austria) and ChemiDoc Touch (BioRad).

**Supplementary figures and table legend:**

**Supplementary figure S1: Validation of meprins antibodies.**


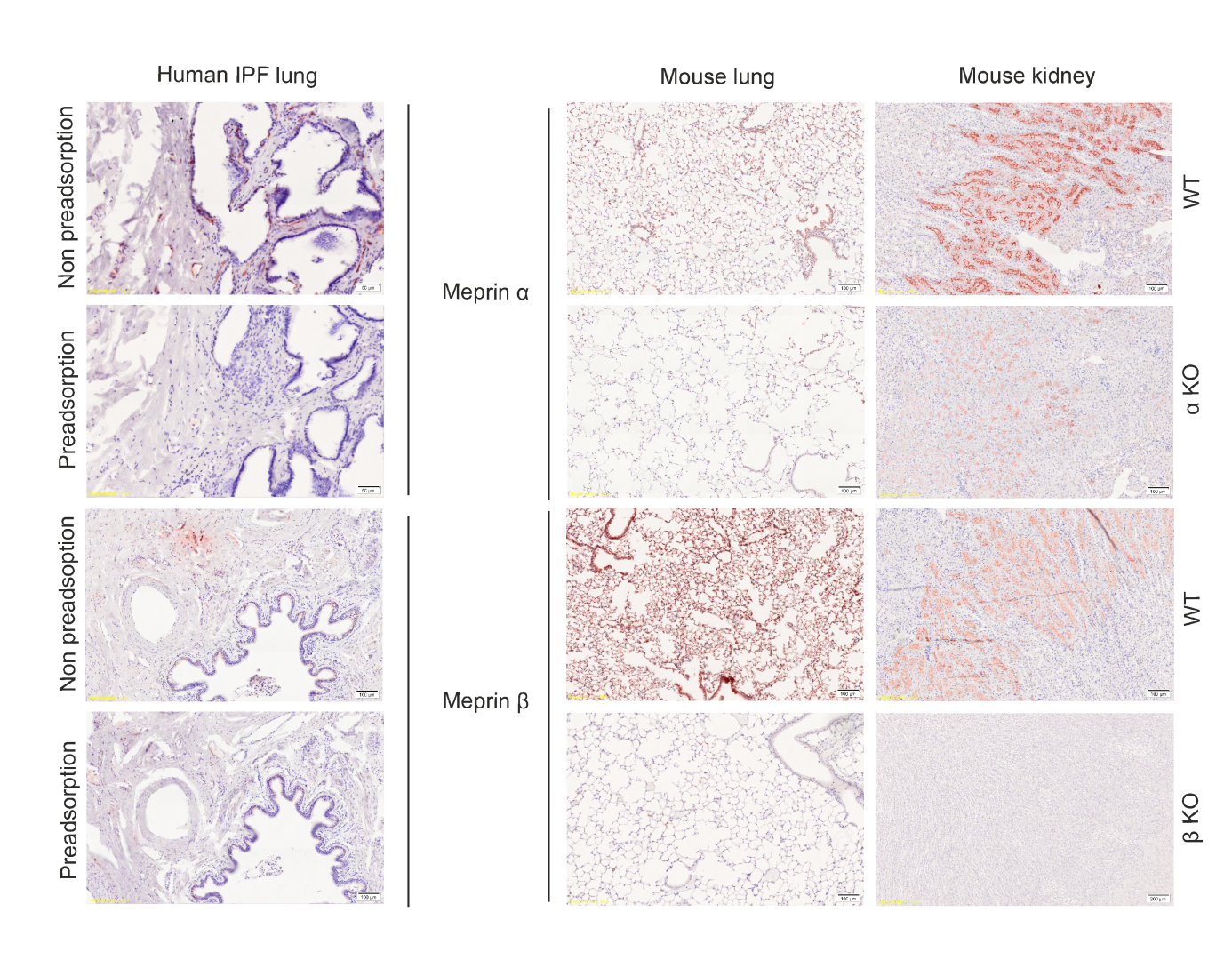


Testing of the human meprins antibodies in human IPF lung with and without pre-adsorption. Testing of the mouse meprins antibodies in mouse lung and kidney from wt littermates and respective meprins KO mice. Scale bars show 100µm.

**Supplementary figure S2: Meprin β is not regulated by short time stimulation of TGF-β and TNF-α in epithelial cells.**


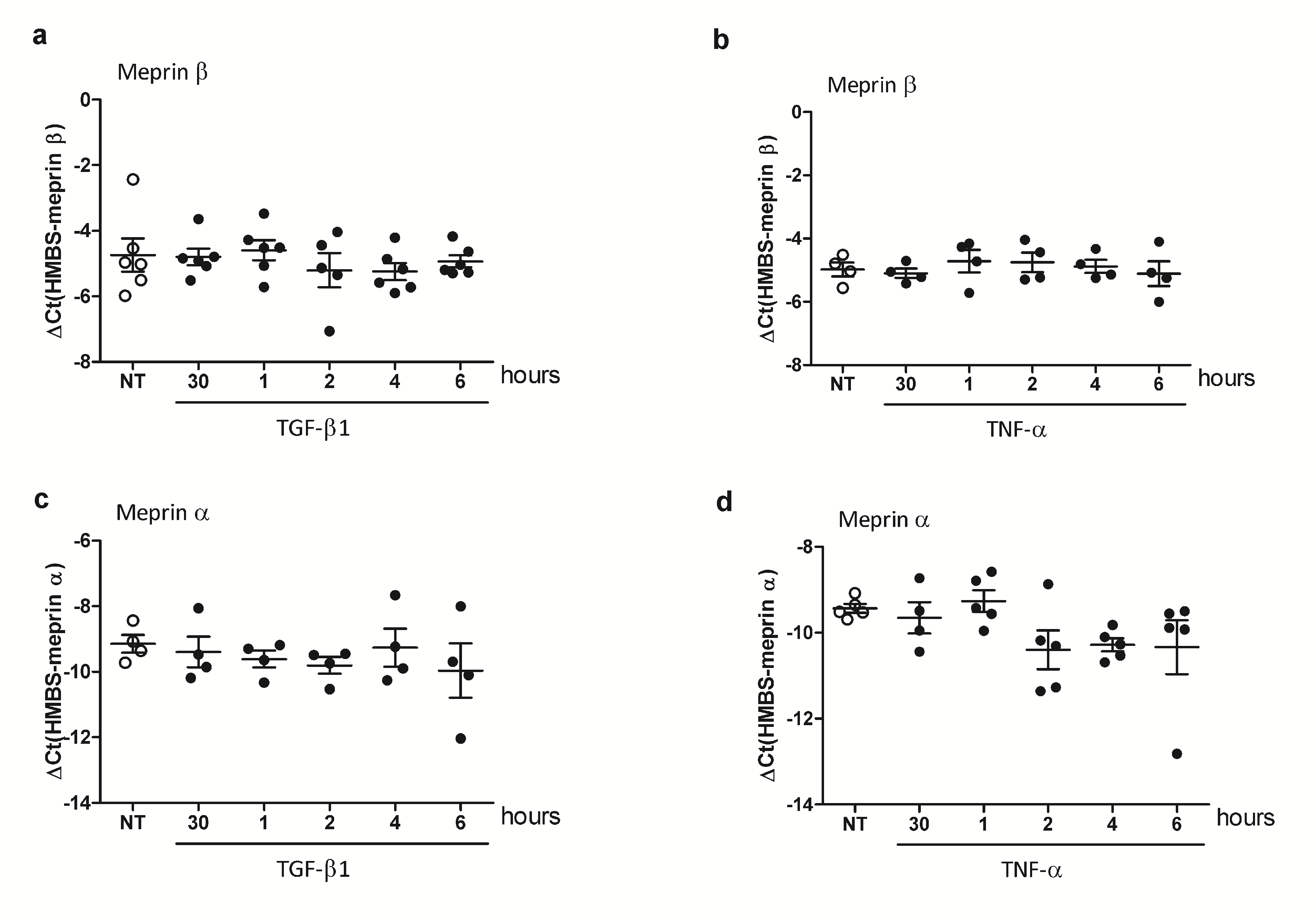
mRNA expression level of meprin β and meprin α upon TGF-β (10 ng/ml) (a, c respectively) and TNF-α (1 ng/ml) (b,d respectively) stimulation on A549 cells for the indicated time points (*p<0.05).

**Supplementary figure S3: Positive controls for permeability assay.**


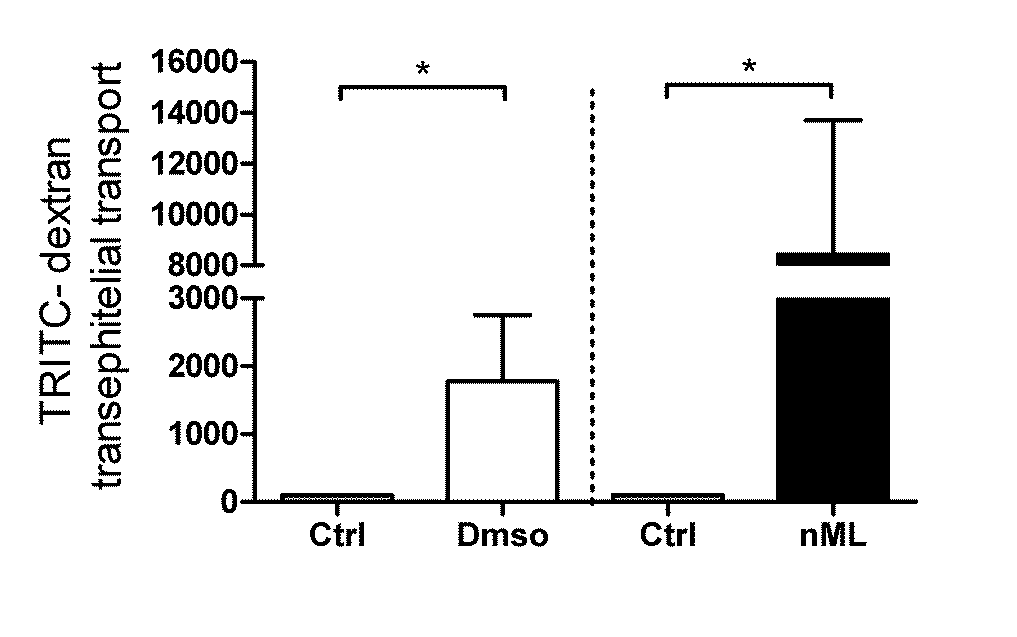


Addition of DMSO as a positive control increased TRITC-dextran permeability of A549 (*p<0.05). Absence of cells (no monolayer=nML) was used as a gain adjustment well for the fluorescence measurement.

**Supplementary figure S4:** **Full length blots for E-cadherin and Lamin**

**
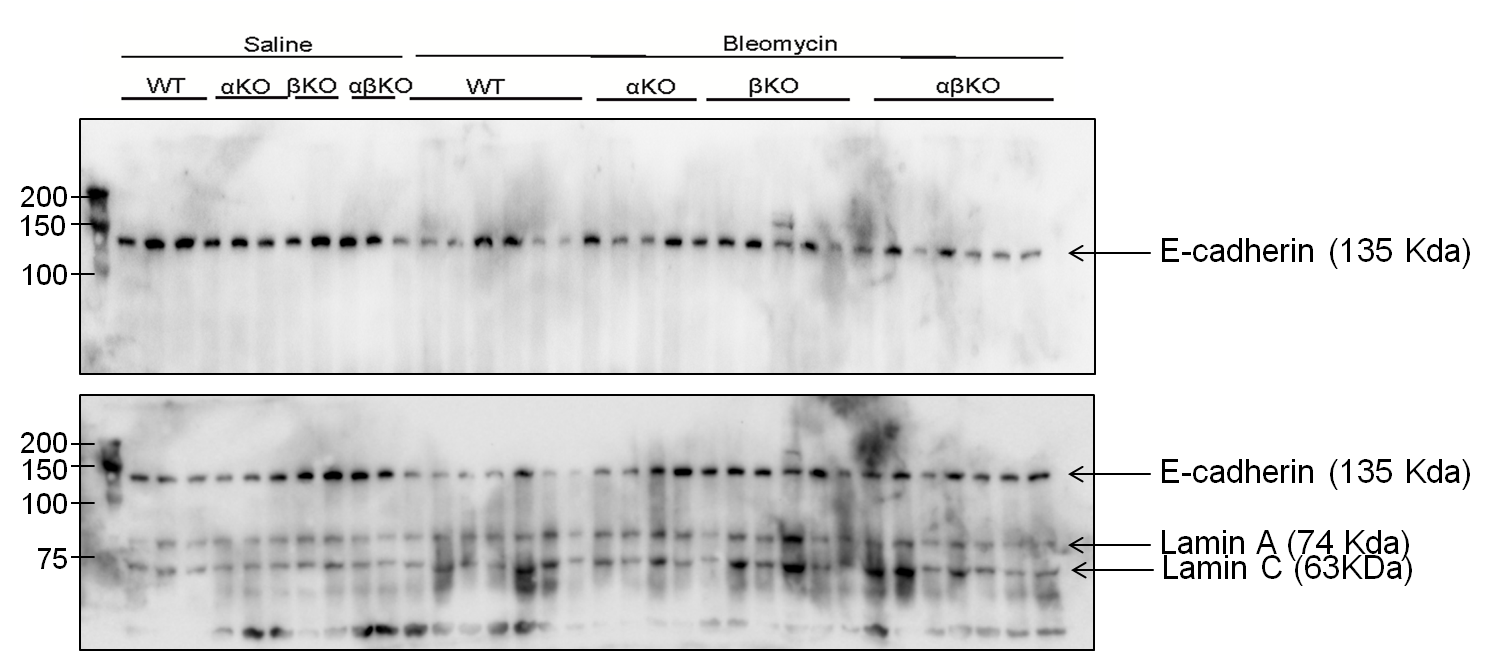
**

Full length blots of E-cadherin and Lamin (after stripping of the membrane) are shown

**Supplementary figure S5:** **Sirius red staining subjected to polarized light revealed less birefringence in bleomycin treated meprins β ko mice in comparison to wt bleomycin challenged mice.**


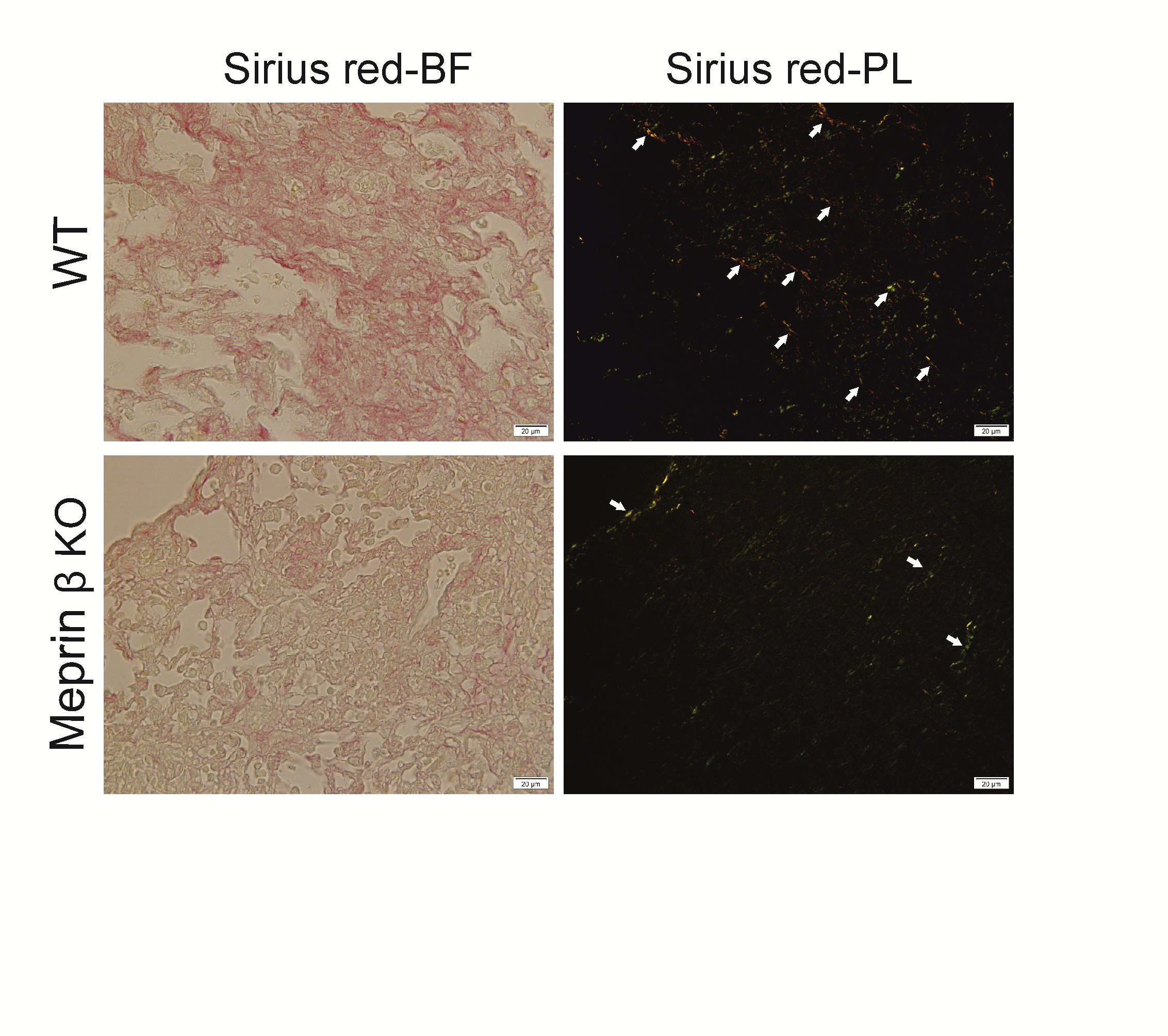


Representative pictures of Sirius red staining under bright field (BF) and polarized light microscopy (PL) of bleomycin treated wt and meprin β KO mice. Arrows point at region with collagen. Scale bars show 20µm.

**Supplementary figure S6:** **Collagen I and III expression level is elevated upon bleomycin treatment.**


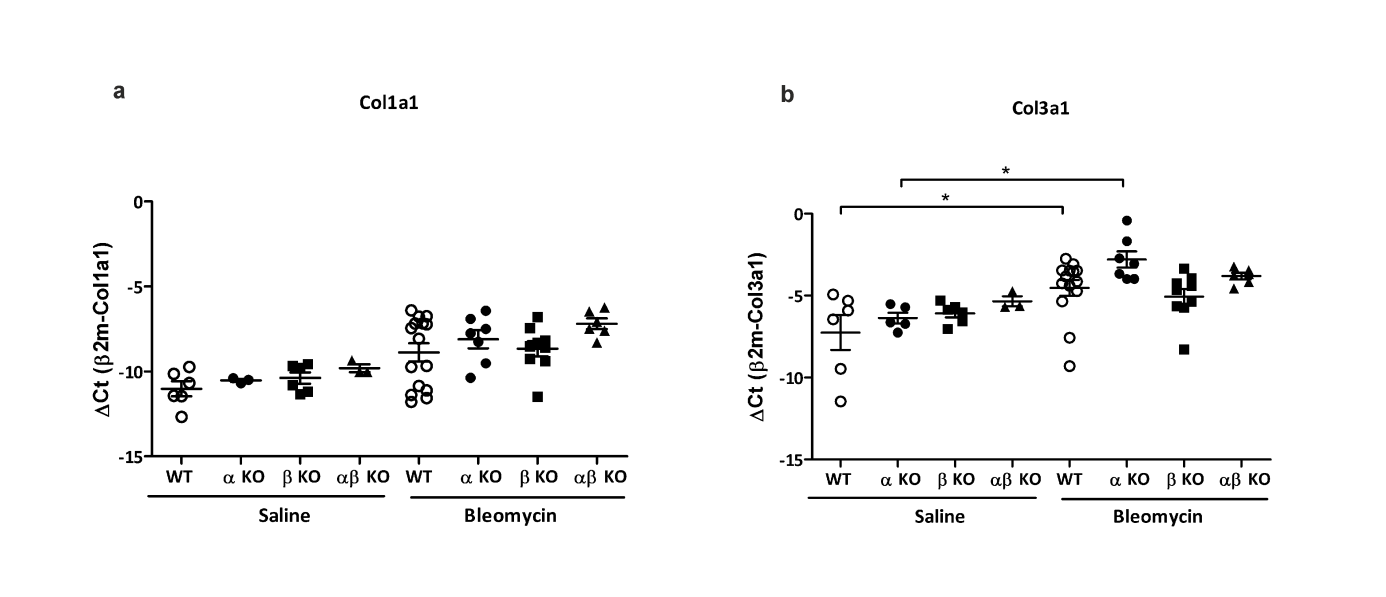
mRNA expression level of a) Collagen I and b) collagen III from lung homogenate of meprin α, meprin β meprin αβ KO and wt littermates mice after 14 days saline or bleomycin treatment (*p<0.05).


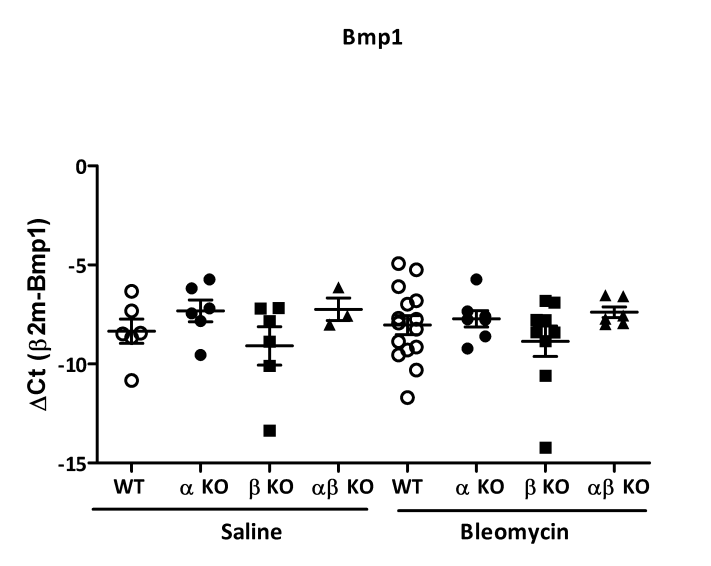
**Supplementary figure S7: BMP-1 mRNA level is not changed in meprins KO mice.**

mRNA expression level of BMP-1 from lung homogenate of meprin α, meprin β meprin αβ KO and wt littermates mice after 14 days saline or bleomycin treatment (*p<0.05).


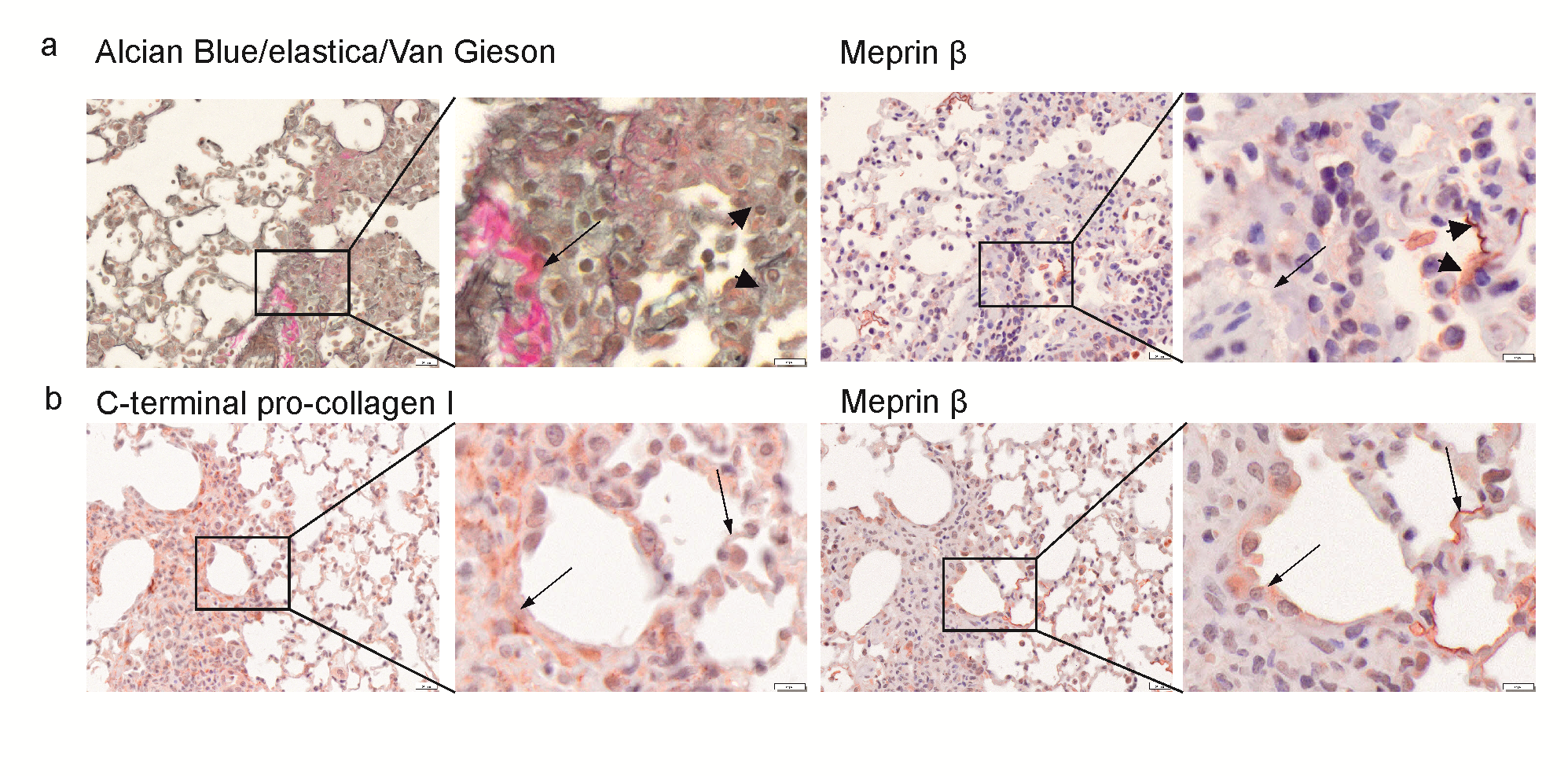
**Supplementary figure S8: Meprin β localize in region of immature collagen and pro-collagen I**

a) Alcian blue/elastica/van Gieson staining and meprin β in serial slides from lung wt mice after 14 days bleomycin treatment. Arrows point at mature collagen (pink) while arrowhead point at immature collagen (greenish grey). e) Representative pictures of immunohistochemical staining in serial slides for C-terminal-pro-collagen I and meprin β. Arrows point at co-localization of meprin β and C-terminal pro-collagen I. Scale bars show 50µm in the overview picture and 10µm in the zoomed area.

**Supplementary table S1**: Sequence of primers

| **Gene** | **Forward Primer (5’-3’)** | **Reverse Primer (5’-3’)** |
| --- | --- | --- |
| Hu MEP1B | CAATTACAGTGGCCATGT | TGTCAGGATTTTGATCCA |
| Hu MEP1A | TGGTGTTCACTACCTCGAA | TATGATAGGTTCCCACCCT |
| HU HMSB | TCGGAGCCATCTGCAAGCGG | GCCGGGTGTTGAGGTTTCCCC |
| Hu B2M | CCTGGAGGCTATCCAGCGTACTCC | TGTCGGATGGATGAAACCCAGACA |
| Mu Col1a1 | | AATGGCACGGCTGTGTGCGA |  | | --- | --- | | AACGGGTCCCCTTGGGCCTT |
| Mu Col3a1 | GCCCTCCCGGGAATAACGGC | TGGCTCTCCCTTCGCACCGT |
| Mu Bmp1 | CCTGTGGTGGATTCCTCACC | TGCACACATCATTGCCCTCA |
| Mu E-cadherin | CCCAGAGACTGGTGCCATTT | TGGCAATGGGTGAACCATCA |
| Mu B2M | CGGCCTGTATGCTATCCAGAAAACC | TGTGAGGCGGGTGGAACTGTG |
